# Astrogliosis has Different Dynamics after Cell Transplantation and Mechanical Impact in the Rodent Model of Parkinson’s Disease

**DOI:** 10.4274/balkanmedj.2016.1911

**Published:** 2018-03-15

**Authors:** Nikola Tomov, Lachezar Surchev, Clemens Wiedenmann, Máté Daniel Döbrössy, Guido Nikkhah

**Affiliations:** 1 Department of Anatomy, Trakia University Faculty of Medicine, Stara Zagora, Bulgaria; 2 Department of Anatomy, Histology and Embryology, Medical University of Sofia, Sofia, Bulgaria; 3 Neurocenter, University Medical Center Freiburg, Freiburg, Germany; 4 Department of Stereotactic Neurosurgery, Friedrich-Alexander University Erlangen-Nürnberg, Erlangen, Germany

**Keywords:** Astroglia, animal model, cell transplantation, experimental, Parkinson’s disease

## Abstract

**Background:**

Transplantation of fetal mesencephalic tissue is a well-established concept for functional reinnervation of the dopamine-depleted rat striatum. However, there is no extensive description of the glial response of the host brain following this procedure.

**Aims:**

The present study aimed to quantitatively and qualitatively analyse astrogliosis surrounding intrastriatal grafts and compare it to the reaction to mechanical injury with the transplantation instrument only.

**Study Design:**

Animal experimentation.

**Methods:**

The standard 6-hydroxydopamine-induced unilateral model of Parkinson’s disease was used. The experimental animals received transplantation of a single-cell suspension of E14 ventral mesencephalic tissue. Control animals (sham-transplanted) were subjected to injury by the transplantation cannula, without injection of a cell suspension. Histological analyses were carried out 7 and 28 days following the procedure by immunohistochemistry assays for tyrosine hydroxylase and glial fibrillary acidic protein. To evaluate astrogliosis, the cell density and immunopositive area were measured in distinct zones within and surrounding the grafts or the cannula tract.

**Results:**

Statistical analysis revealed that astrogliosis in the grafted striatum increased from day 7 to day 28, as shown by a significant change in both cell density and the immunopositive area. The cell density increased from 816.7±370.6 to 1403±272.1 cells/mm^2^ (p<0.0001) аnd from 523±245.9 to 1164±304.8 cells/mm^2^ (p<0.0001) in the two zones in the graft core, and from 1151±218.6 to 1485±210.6 cells/mm^2^ (p<0.05) for the zone in the striatum immediately adjacent to the graft. The glial fibrillary acidic protein-expressing area increased from 0.3109±0.1843 to 0.7949±0.1910 (p<0.0001) and from 0.1449±0.1240 to 0.702±0.2558 (p<0.0001) for the same zones in the graft core, and from 0.5277±0.1502 to 0.6969±0.1223 (p<0.0001) for the same area adjacent to the graft zone. However, astrogliosis caused by mechanical impact only (control) did not display such dynamics. This finding suggests an influence of the grafted cells on the host’s glia, possibly through cross-talk between astrocytes and transplanted neurons.

**Conclusion:**

This bidirectional relationship is affected by multiple factors beyond the mechanical trauma. Elucidation of these factors might help achieve better functional outcomes after intracerebral transplantation.

Ever since the first experiments with intracerebral transplantation of nervous tissue, the graft-host interface has been recognised as a site at which important processes of interaction and integration occur between the transplanted cells and the host brain (1,2,3). Astroglia play a major role in the events that occur at this interface. Astrocytes of the host brain tissue not only delineate the grafts, but also show activation some distance from their borders. Introduction of the transplantation instrument also induces activation of the astroglia (4) due to tissue trauma. Even though the gliotic reaction to both interventions is similar, no comparison between the relative contribution of transplanted cells and trauma to astroglial scarring has been undertaken until now.

Reducing tissue injury during transplantation in Parkinson’s disease is shown to reduce glial activation. At the same time, the extent of glial scarring surrounding the graft is negatively correlated with the number of integrated neurons (5). This correlation, however, has not been proven to be causative. It is not clear if more pronounced astrogliosis leads to poorer neuronal integration, or whether both events occur secondary to greater tissue trauma. On one hand, the glial scar could act as a mechanical barrier for neurites, preventing them from reinnervating the host brain. This has been demonstrated in a number of spinal trauma experiments (6,7,8,9). However, astrocytes provide support for outgrowing axons (2). Glial scar tissue is known to contain growth-promoting molecules such as laminin and fibronectin (10). Any disruption might deprive the neurons of this support, thereby exacerbating the tissue injury (11). Despite there being numerous reports in the literature focussed on central nervous system (CNS) trauma, the glial reaction to grafting is poorly understood. Specifically, it is not clear how the glial scar tissue surrounding the grafts interacts with the transplanted cells. Moreover, there has been no extensive description of astrogliosis in response to intracerebral transplantation in a model of Parkinson’s disease. The aim of the present study was to analyse the astrocytic reaction following transplantation of embryonic mesencephalic tissue in a model of Parkinson’s disease. This study provides quantitative and qualitative descriptions of the astroglial reaction to grafting and compares it with the reaction following a mechanical injury only.

## MATERIALS AND METHODS

**Figure 1:**
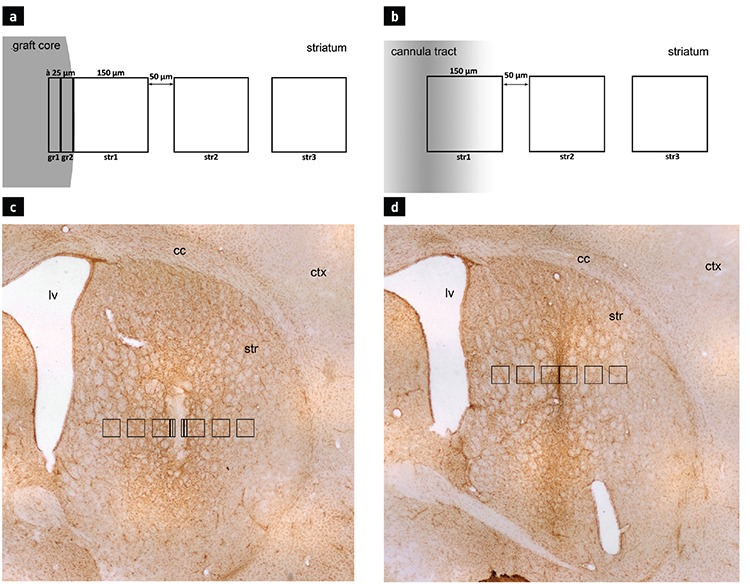
Measurement zones in grafted animals (a,c) and sham-transplanted animals (b,d). The graft core (a) and the cannula tract (b) are indicated on the left-hand side of the graphical representation. *Str: striatum; cc: corpus callosum; ctx: cortex; lv: lateral ventricle*

A total of 31 adult male Sprague-Dawley rats (body weight 300-350 g) were used for this study. The animals were kept under standard conditions (12/12 h dark/light cycle, *ad libitum *access to food and water) until sacrifice. The study was approved by the veterinary board for animal research of the University of Freiburg (TVA G-10/110) and was carried out in accordance with the European Union (EU) Directive 2010/63/EU concerning the protection of animals used for scientific purposes. All experimental animals were subjected to a unilateral 6-hydroxidopamine-induced lesion of the nigrostriatal pathway according to Ungerstedt (12). This was achieved by injecting a 3.6% 6-hydroxydopamine solution stabilised with approximately 0.2 mg/mL ascorbic acid into the medial forebrain bundle. The effect of the lesion was confirmed 28 days afterwards via amphetamine-induced rotational behaviour testing, in which only animals that exhibited more than seven rotations ipsilateral to the lesion were included in the experiment (13). The transplantation was carried out using a single-cell suspension of ventral midbrain tissue, obtained from embryos at E14 and processed according to a standard procedure (14). Using a Hamilton syringe with a 26 gauge steel cannula, two deposits (approximately 100.000 cells each) were transferred into the rat striata according to Paxinos and Watson (15) at the following stereotaxic coordinates: anteroposterior (AP), 0.2; laternal, 3.5; dorsoventral, -5.0 and 4.0. Sham-transplanted animals were subjected to penetration of the brain parenchyma at the same coordinates using the transplantation cannula, but without injecting anything. Animals were sacrificed on day 7 or day 28 following the surgical procedure. After transcardial perfusion with 4% paraformaldehyde, the rat brains were sectioned in the coronal plane on a freezing microtome at 40 µm thickness. The resulting serial free-floating sections were then processed for immunohistochemistry using primary antibodies against tyrosine hydroxylase (TH) and glial acidic fibrillary acidic protein (GFAP). The reaction was visualised using 3.3-diaminobenzydine as a chromogen. For the image analysis, all sections containing a graft were used. Grafts were found at AP coordinates between 0.7 and -0.4 relative to the bregma. Due to this considerable size, at least three sections were used from each animal. Evaluation of astrogliosis surrounding the grafts or the impact of injury by the transplantation cannula in the sham group were carried out by measuring two parameters, cell density and the immunopositive area. The measurements were performed in three areas of the surrounding tissue of the host’s striatum for both transplanted (Figure 1a-1d) and sham-transplanted animals (Figure 1b), according to the scheme by Jonas et al. (16). These two parameters were also measured in two areas of the graft core (for the animals receiving a transplant). Those two areas (each 25-µm wide) covered the periphery of the grafts containing the most prominent area of astrogliosis adjacent to the graft-host interface, and further divided it into two areas, an inner and outer zone. Moreover, care was taken to analyse zones located both medially and laterally to the graft. Therefore, at least six measurements were carried out for each animal, consisting of areas medial and lateral to the graft for all of the graft-containing sections. For image analysis, digital images were obtained using a Nikon Eclipse 80i microscope equipped with a DMX 1200c camera (Nikon Instruments Europe, Netherlands). The cell density was determined by manually counting visible cell bodies in the respective areas. The immunopositive area was measured as a fraction of grey pixels with values above a predefined threshold from all pixels within the area examined. This threshold was determined by applying Otsu’s algorithm (17) on an image of the contralateral intact striatum for the same coordinates.

### Statistical analysis

Data were analysed using GraphPad Prism 6 for Windows (GraphPad Software, Inc., USA). One-way analysis of variance (ANOVA), which is normally used for comparing three or more groups of ordinary data measures, was applied, followed by Tukey-Kramer’s post-hoc test for multiple comparisons. This test was chosen because the generated p-values tended to be more liberal than the ones given by Bonferroni’s post-hoc test, but more conservative than those generated by the Neuman-Keuls’ test. To avoid introducing errors, the raw data from each measurement was used in the analyses without any preliminary averaging. P-values less than 0.05 were considered statistically significant. All data hereafter is presented as mean values ± standard deviation.

## RESULTS

**Figure 2:**
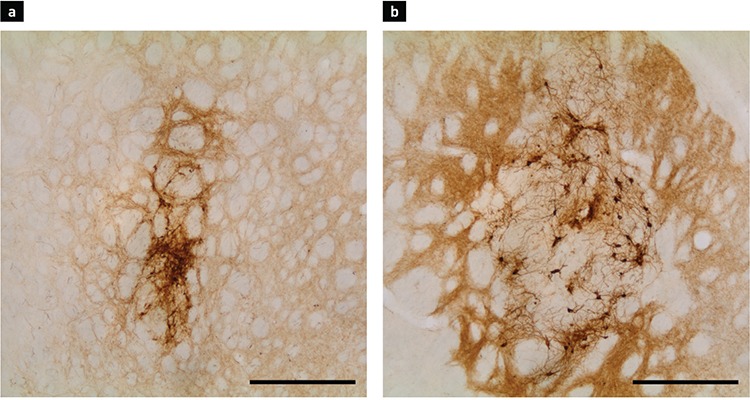
Tyrosine hydroxylase staining of intrastriatal grafts on day 7 (a) and day 28 (b) following transplantation. The scale bar represents 500 µm.

**Figure 3:**
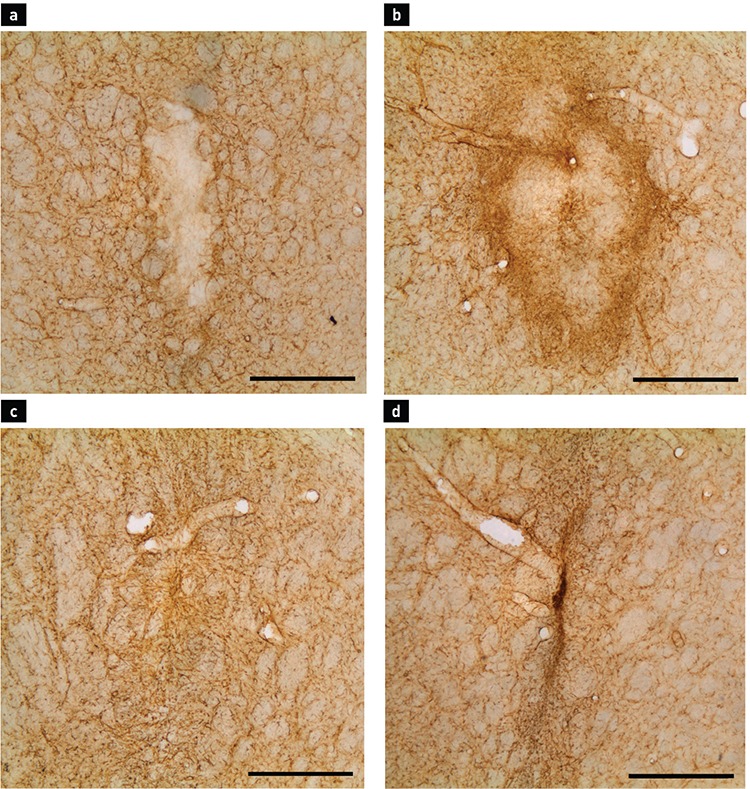
Glial fibrillary acidic protein staining of the striatum. Intrastriatal graft on day 7 (a) and day 28 (b) following transplantation. Cannula tracts on day 7 (c) and day 28 (d) following the mechanical injury. The scale bar represents 500 µm.

In all animals from the groups that received a cell suspension injection, TH+ cells were visible in the striatum at all coordinates (Figure 2a, 2b). Perikarya were gathered in clusters, mainly associated with the graft-host interface. Some processes were seen to be emerging from the cell bodies and penetrating the denervated tissue of the host striatum on day 7 after transplantation. On day 28, those processes reached greater lengths. In addition, diffuse restoration of normal TH immunoreactivity of the striatum was observed around the grafts. Contrary to this finding, no TH immunoreactivity was observed in the animals subjected to mechanical injury only, with the right striata remaining completely unstained. On day 7 after the transplantation procedure, numerous GFAP+ protoplasmic astrocytes were visible surrounding the grafts (Figure 3a). Astroglial cells were observed delineating the graft with their processes, sometimes sending longer projections inside the graft core. Intensively stained protoplasmic astrocytes were found in the host tissue some distance from the graft-host interface. As the distance from the transplant increased, these were gradually replaced with the resident astroglia of the striatum. In contrast to that observed in the host tissue, only a few GFAP+ structures were visible in the graft core.

**Figure 4:**
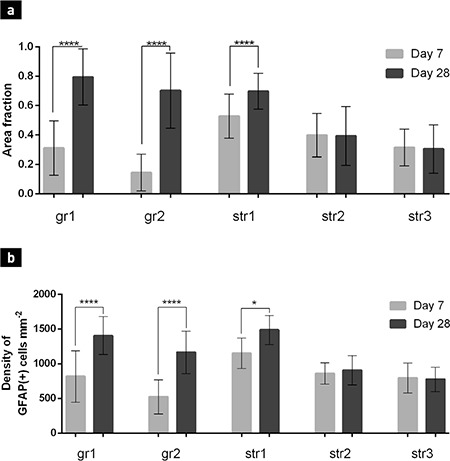
Dynamics of the immunopositive area (a) and cell density (b) in transplanted animals between day 7 and day 28 after transplantation. Data are presented as mean values ± standart deviation (*p<0.05; ****p<0.0001). *str: striatum; GFAP: glial fibrillary acidic protein*

**Figure 5:**
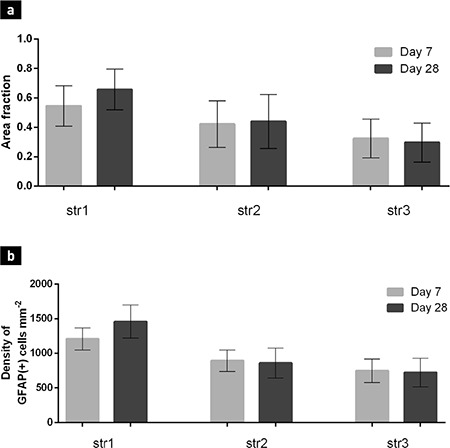
Dynamics of the immunopositive area (a) and cell density (b) in sham-transplanted animals between day 7 and day 28 after the procedure. Data are presented as mean values ± standart deviation. There was no significant change between day 7 and day 28. *str: striatum; GFAP: glial fibrillary acidic protein*

The appearance of the astrocytes surrounding the grafts changed from day 7 to day 28 after transplantation. Their bodies could be described as having become smaller with an irregular shape. Their processes tended to be shorter and thicker. Astrocytes immediately adjacent to the graft formed an optically dense GFAP+ network enveloping the whole transplant (Figure 3b). They were observed to have penetrated the graft-host interface with their projections and to have connected with astrocytes inside the graft core. Some parts of this astroglial envelope showed a tendency to contain processes, which were positioned radially to the graft. When compared with sections stained for TH, the same regions contained TH+ fibres directed parallel to the radial GFAP+ fibres. In the graft core, some clusters of astroglia with radially directed processes were also found. A characteristic finding on day 28 was newly formed blood vessels that were surrounded by astrocytes. Some vessels passed through the graft core, while others crossed the graft-host interface. The mechanical impact on the striatum appeared to activate the astrocytes along the cannula tract, similar to the activation observed around grafts. However, due to the absence of a transplant, the tissue surrounding the tract was not displaced nor compressed. Astrogliosis in these animals manifested as a vertical band of small protoplasmic astrocytes in the striatum (Figure 3c, 3d). The innermost part of this band, corresponding to the cannula tract itself, consisted of densely arranged astroglial cells. Medially and laterally, the reaction became less evident as the number of protoplasmic astrocytes decreased with greater distance. Statistical analysis of the GFAP+ cell density (Table 1) and immunopositive area (Table 2) showed a marked increase in both parameters from day 7 to day 28 in animals that received the graft (Figure 4a, 4b). The graft and striatum in the zone in immediate proximity to the graft-host interface showed an increased absolute number of astrocytes in the given time frame (816.7±370.6 vs. 1403±272.1 cells/mm^2^, p<0.0001 for gr1; 523±245.9 vs. 1164±304.8, p<0.0001 for gr2; 1151±218.6 vs. 1485±210.6, p<0.05 for str1). Aside from the greater number of cells, the GFAP-expressing area of the tissue was also increased (0.3109±0.1843 vs. 0.7949±0.1910, p<0.0001 for gr1; 0.1449±0.1240 vs. 0.702±0.2558, p<0.0001 for gr2; 0.5277±0.1502 vs. 0.6969±0.1223, p<0.0001 for str1). This was in accordance with the described formation of the astroglial envelope around the graft. Astroglia in the zones further away from the transplant did not show such dynamics. Around the cannula tract in the brains of sham-transplanted animals, the measured parameters tended to remain unchanged from day 7 to day 28 (Figure 5a, 5b). Furthermore, the cannula injury did not appear to induce the formation of an astrocytic envelope around the traumatic zone like that visible around the grafts.

## DISCUSSION

Astrocytes of the glial scar support and protect the transplanted neurons (18). They also produce certain components of the tissue that forms around grafted cells, facilitating neurite outgrowth (10). Astrogliosis is an important regulator of CNS restoration following traumatic impact, stimulating trophic effects and neovascularisation. On the other hand, gliosis may hinder regeneration as the mechanical barrier of the gliotic scar is generally considered impenetrable for axons (6,7,8,9,19,20). The findings of the present study suggest that the formation of a glial scar around transplanted to the CNS neurons is a complex process involving multiple factors. It is clear that traumatic injury to the brain parenchyma by the transplantation instrument induces gliosis (5). However, for the first time, we quantitated the extent that gliosis is modified by the presence of transplanted cells. Stimulation by the grafts appears to be more significant for astrocytes than the influence of the trauma alone. One proposed mechanism may be direct neuron-glia crosstalk. It is known that astrocytes possess neurotransmitter receptors and ion channels in addition to secreting so-called “gliotransmitters” (21,22,23). Combined with the fact that glial cells are able to detect zones of increased neuronal activity (24,25), this suggests that transplanted cells and their activity during integration with the host brain can actively influence astrocytes.

In the context of neural transplantation, it is known that astrocytes are active supporters of reinnervation of the host brain by the graft. It has been previously reported that reactive astrocytes of the host brain direct their processes in a direction parallel to the outgrowing axons (26), consistent with that observed in the current study. Moreover, specific organisation of astrocytic membranes to provide a scaffold for axons is needed for the processes of transplanted neurons to be able to penetrate and grow beyond the glial scar (27). The grafted neural cells are not only a passive beneficiary of this astroglial support, but can also directly stimulate astrogliosis. The processes of synaptic integration between the graft and host can induce astrocytic proliferation and migration (24,25). In this way, transplanted cells actively induce a gliotic reaction, providing the metabolic and structural support required for their functioning, and also facilitating new synapse formation. Another aspect of astrogliosis surrounding grafts is the vascularisation of transplanted tissue. As the injected suspension contains only single, dissociated cells, it is completely avascular. Therefore, integration of the graft with the host brain relies on the formation on a vascular bed to nourish the transplanted cells. The influence of trauma on the nervous tissue is itself a stimulus for the formation of vessels within the tissue of the glial scar (28). Formation of the basal lamina of the newly formed endothelium of this vascular bed is closely associated with astroglial activity (29). This is best demonstrated on a morphological level by the dense GFAP+ envelope surrounding the blood vessels. Those astrocytic elements are predominantly derived from striatal astrocytes, but can also result from the perivascular migration of astrocytes from the graft towards the host brain (30). Reactive astrogliosis following transplantation of neural tissue to the CNS is a continuous process that extends beyond the transplantation event itself. Astrocyte recruitment along the graft-host interface is more pronounced than that caused by analogous tissue injury. The process is clearly influenced by the transplanted cells. Reinnervation of the host brain by the graft could induce the formation of a glial scaffold, which supports the outgrowing axons of the transplanted cells. Furthermore, the vascularisation of the graft as a part of its integration with the host brain is a process that requires the active participation of astrocytes. Therefore, the astroglial scar is not merely a mechanical envelope around foreign tissue, but rather an active provider of functional and structural support. The cross-talk between the transplanted neurons and glia can potentially influence functional recovery, and should be discussed in the context of neuronal replacement therapy.

## Figures and Tables

**Table 1:**
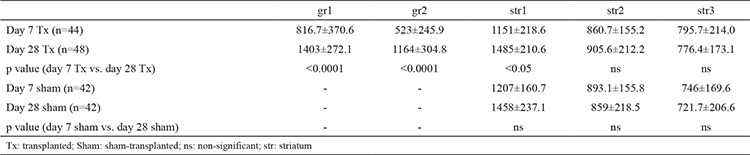
Analysis of positive glial fibrillary acidic protein cell density (cells per mm^2^) measurements in different zones. Data are presented as mean values ± standart deviation

**Table 2:**
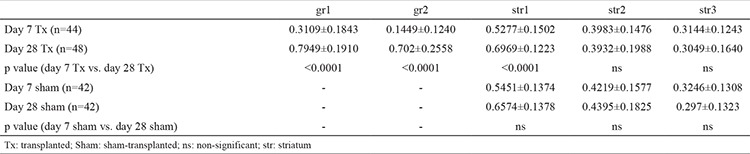
Analysis of immunopositive glial fibrillary acidic protein area measurements in different zones. Data are presented as mean values ± standart deviation
